# Seed-dressing treatments improve wheat germination and seedling growth under contrasting sowing conditions in a rice–wheat rotation system

**DOI:** 10.3389/fpls.2026.1970127

**Published:** 2026-10-29

**Authors:** Yang Gao, Yapeng Li, Yong Ye, Meiling Li, Xiulan Huang, Qiaojia Zhao, Qi Sui, Haiyan Zhang, Wei Li, Hongkun Yang, Gaoqiong Fan

**Affiliations:** 1 College of Agronomy, Sichuan Agricultural University, Chengdu, Sichuan, China; 2 Key Laboratory of Crop Eco-physiology and Farming System in Southwest China, Ministry of Agriculture and Rural Affairs, Chengdu, Sichuan, China

**Keywords:** emergence dynamics, root architecture, seed-dressing agent, sowing method, wheat, yield

## Abstract

**Introduction:**

Delayed sowing after rice harvest increasingly compromises wheat establishment and yield formation across rice–wheat rotation regions. Seed dressing may alleviate these constraints, but its effectiveness under humid, late-sown conditions and contrasting soil-cover practices remains insufficiently understood.

**Methods:**

A two-year, two-factor split-plot field experiment was conducted in Sichuan Province, China. Soil-covered and uncovered surface sowing were assigned to the main plots, and eight seed-dressing regimes were assigned to the subplots. Treatments included Coolas, brassinolide, and Ju Chuan, applied individually or in combination. Germination traits, field emergence, seedling morphology, root architecture, population growth, yield components, and grain yield were evaluated.

**Results:**

Seed-Dressing Treatments significantly improved seed germination and early stand establishment. Compared with the untreated control (CK), Ju Chuan combined with brassinolide and half-rate Coolas (T_J+B+50%C_) increased germination percentage, germination index, and vigor index by 12.89%, 96.12%, and 22.88%, respectively. Coolas alone (T_C_) produced the greatest improvement in seedling vigor index, which was 97.69% higher than that of CK. T_C_ also promoted root system development, crop growth rate, and dry matter accumulation. Soil cover shortened field emergence time and increased emergence percentage. Treatment responses varied across developmental stages, with brassinolide alone (T_B_) primarily accelerating field emergence. By contrast, T_C_ consistently increased grain yield by 12.5%–21.3% across both growing seasons and sowing methods.

**Discussion:**

Seed dressing can partially offset late-sowing constraints by improving germination, emergence, root development, stand establishment, and subsequent yield formation. Under the tested field conditions, several seed-dressing treatments showed favorable agronomic responses with soil-covered sowing. However, the study was limited to one cultivar at a single site over two growing seasons. Broader recommendations therefore require validation across multiple cultivars, locations, and agroecological conditions.

## 1 Introduction

Wheat (Triticum aestivum L.) is a major winter crop in rice–wheat rotations, supporting food security and efficient annual land use (Kettlewell et al., 2023; Dhanda et al., 2022; Nawaz et al., 2019). Climate change is reshaping rainfall patterns and increasing heavy precipitation and flooding across many wheat-producing regions (Intergovernmental Panel on Climate Change, 2021). This risk is acute in rice–wheat systems because wheat must be sown soon after rice harvest within a narrow window. At this stage, paddy soils often remain wet and poorly aerated because of recent flooding, a compact plough layer, and limited drainage. Rainfall during rice harvest or wheat sowing can saturate seedbeds, delay field operations, and expose germinating seeds to waterlogging stress (Kanjevac et al., 2023; Zaman et al., 2015; Ding et al., 2021; Du et al., 2022; Liu et al., 2022). Waterlogging displaces air from soil pores and restricts oxygen diffusion, creating hypoxic or anoxic conditions around germinating seeds and young roots. Oxygen deficiency limits mitochondrial respiration and ATP production, impairing endosperm reserve mobilization and the elongation of radicles and coleoptiles (Herzog et al., 2016). In the field, these effects cause delayed, uneven emergence, a lower emergence percentage, weak seedlings, and restricted seminal root growth. Prolonged hypoxia also promotes fermentation, disrupts ion transport and nutrient acquisition, and alters the hormonal regulation of root development. Reactive oxygen species may accumulate during waterlogging and re-aeration, leading to membrane lipid peroxidation and cellular damage (Feng et al., 2022). Together, these processes reduce root activity, leaf growth, dry matter accumulation, tiller initiation, and tiller survival. They ultimately constrain spike formation and yield, particularly when waterlogging coincides with late sowing and low temperature (Lollato et al., 2024; Afzal et al., 2020; Anwar et al., 2024; Bugingo et al., 2025; Cardoso et al., 2021).

Pre-sowing seed dressing offers a practical intervention when wheat is most vulnerable to soil waterlogging and biological stress. Conventional formulations contain fungicides or insecticides that protect seeds and young seedlings from seed-borne and soil-borne pathogens and soil-dwelling pests. Such protection is especially important in wet seedbeds, where slow emergence prolongs pathogen exposure (Paulikienė et al., 2025; Brown et al., 2021; Ayesha et al., 2021; van Herk et al., 2020). Functional formulations may also contain plant growth regulators, micronutrients, microbial inoculants, and biostimulants. These components may activate metabolism, promote reserve mobilization, strengthen antioxidant defense, stabilize cellular membranes, and regulate hormone-mediated root initiation (Zaimbashi et al., 2025; Hoseini et al., 2022; Qiu et al., 2020; Asaduzzaman et al., 2021; Cardoso et al., 2021). Micronutrients contribute to enzyme function and early seedling nutrition, while microbial inoculants and biostimulants may enhance rhizosphere activity, root growth, and nutrient acquisition. These functions may improve emergence rate and uniformity, seedling vigor, and recovery after transient waterlogging. However, treatment efficacy depends on formulation, dose, seed quality, soil temperature, waterlogging intensity and duration, soil properties, and local pest and disease pressure (Pedrini et al., 2017). Previous studies have generally focused on individual early-stage traits, such as germination, emergence, or seedling growth, often under controlled conditions (Lollato et al., 2024). Field evidence remains limited on whether these early gains persist through tillering and contribute to stable yield formation under late-sown, waterlogging-prone conditions.

We therefore conducted a field study of functional seed-dressing treatments under late-sown, waterlogging-prone conditions in a rice–wheat rotation system. Treatments were compared from germination and field emergence through tillering and yield formation. We measured seed germination, emergence dynamics, seedling morphology, root development, dry matter accumulation, tiller formation, and yield-related traits. The study evaluated the functional profiles and agronomic suitability of the treatments under wet field conditions. It also tested whether improvements in establishment, roots, and seedling vigor were associated with sustained crop development and more stable yield formation. Linking early establishment traits with later agronomic performance may provide an evidence base for selecting seed-dressing treatments in rice–wheat rotation regions. The findings may aid reliable wheat establishment amid increasingly variable rainfall and recurrent waterlogging risk.

## 2 Materials and methods

### 2.1 Experimental site

The experiment was conducted from 2024 to 2026 at the Modern Grain Industry Park in Dayi County, Chengdu, Sichuan Province, China (103°53′E, 30°58′N). The site is located in a low-hill region and belongs to the subtropical humid monsoon climate zone. The soil type is loam. Before sowing, the 0–20 cm soil layer contained 38.97 g kg^-1^ organic matter, 1.89 g kg^-1^ total nitrogen, 1.13 g kg^-1^ total phosphorus, 18.53 g kg^-1^ total potassium, 21.02 mg kg^-1^ available phosphorus, 87.70 mg kg^-1^ available potassium, and 248.19 mg kg^-1^ alkali-hydrolysable nitrogen, with a pH of 7.15. The preceding crop at the experimental site was rice.

Meteorological data during the experimental period are shown in **Figure 1**.

**Figure 1:**
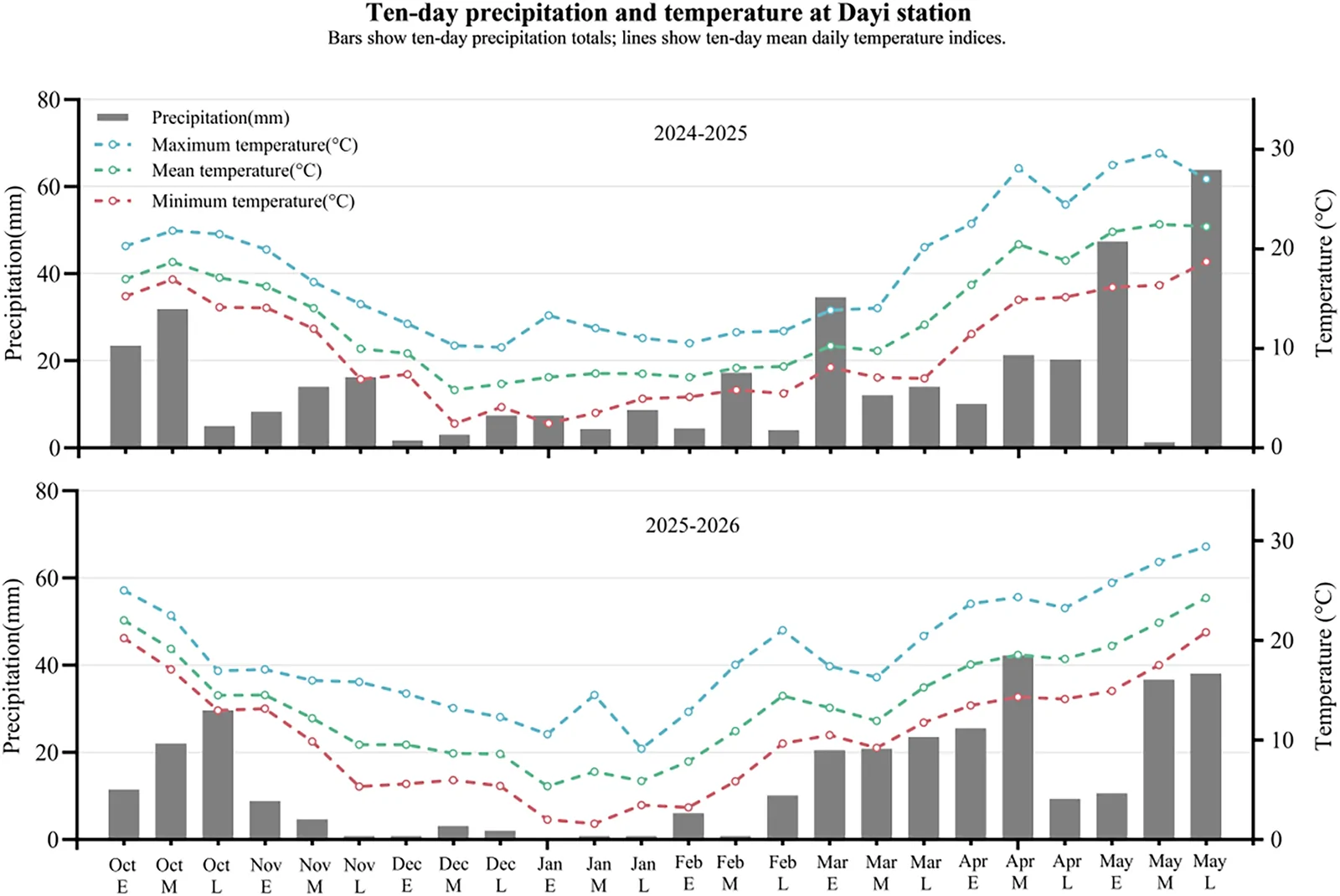
Variations of air temperature and precipitation during the early wheat growth period.

### 2.2 Seed treatments and experimental design

The wheat cultivar Shumai 1963, developed by the Wheat Research Institute of Sichuan Agricultural University, was used in this study. Three commercial products were evaluated as seed-treatment agents. Brassinolide (BR; 0.01% active ingredient) was supplied as an aqueous solution by Shanghai Lvze Biotechnology Co., Ltd. (pesticide registration No. PD20130042). Ju Chuan (JC; Chengdu Jijian Biotechnology Co., Ltd.) was declared to contain macronutrients, silicon, titanium, secondary and trace elements, seaweed polysaccharides, and alginic acid. JC was an organic aqueous formulation with trademark application/registration No. 91694149. It was not a pesticide formulation and therefore had no pesticide registration number issued by China’s Ministry of Agriculture and Rural Affairs. Coolas (Syngenta Group Co., Ltd.) was supplied as a flowable concentrate for seed treatment (pesticide registration No. PD20161551) and contained 22.6% thiamethoxam, 2.2% fludioxonil, and 2.2% difenoconazole. The field experiment followed a two-factor split-plot design with three replicates. The main-plot factor comprised soil-covered and uncovered surface sowing, whereas eight seed-treatment regimes were assigned to the subplots. The untreated control was designated CK. Single-agent treatments were T_C_ (Coolas), T_B_ (BR), and T_J_ (Ju Chuan). Combined treatments were T_C+B_ (Coolas + BR), T_J+50%C_ (Ju Chuan + half-rate Coolas), T_J+B_ (Ju Chuan + BR), and T_J+B+50%C_ (Ju Chuan + BR + half-rate Coolas). Treatment dosages were expressed on a seed-mass basis. Coolas, Ju Chuan, and brassinolide were applied at 2.0, 8.0, and 3.0 mL kg^–1^ seed, respectively. Half-rate Coolas was applied at 1.0 mL kg^–1^ seed. For each treatment, product concentrations and application rates were identical across sowing methods and growing seasons. Each treatment formulation was mixed with water at a water-to-seed ratio of 1:50 (w/w). Seed dressing was performed using a 5BYX-5 continuous-flow seed-coating machine (Shijiazhuang Sanli Grain Machinery Co., Ltd., Shijiazhuang, Hebei, China). Coating uniformity was confirmed visually by uniform seed coloration, formation of a continuous and intact film over the seed surface, and absence of localized peeling or flaking. The coated seeds were air-dried in the shade for 24 h before sowing. The 2 × 8 factorial arrangement yielded 16 treatment combinations and 48 experimental plots, each covering 48 m^2^. Field experiments were conducted in Dayi County, Sichuan Province, China, during the 2024–2025 and 2025–2026 growing seasons. Sowing took place on 6 November 2024 and 10 November 2025 using a Yongxiang precision grain seeder with a row spacing of 12 cm. Two sowing methods were compared: soil-covered sowing, in which seeds were covered with approximately 5 cm of soil, and surface sowing, in which seeds were left uncovered on the soil surface. Seeding rates were 225 kg ha^–1^ in 2024–2025 and 300 kg ha^–1^ in 2025–2026. Within each growing season, the seeding rate was identical across both sowing methods and all eight seed-treatment regimes. The higher seeding rate in the second season was adopted to support adequate seedling establishment under wetter field conditions at sowing.

A compound fertilizer (N–P–K, 15–15–15) was applied as basal fertilizer, and urea (46.0% N) was used for topdressing. Nitrogen, phosphorus, and potassium were applied at 180, 90, and 90 kg ha^–1^, respectively. Half of the total N and all P and K were applied before sowing. The remaining N was divided equally between the two-leaf stage with the third leaf emerging and the jointing stage. Fertilizers were surface-broadcast, and all other crop-management practices were applied uniformly across treatments according to local wheat-production practices.

### 2.3 Determination of germination-related indicators

Laboratory germination experiments were conducted for the 2024–2025 and 2025–2026 growing seasons using seeds from the same batches and subjected to the same seed-dressing regimes as those used for the corresponding field sowings. Year labels for the laboratory experiments indicate the actual calendar year of measurement. Two layers of sterilized filter paper were placed in sterilized Petri dishes with a diameter of 9 cm. The filter paper was initially moistened with 30 mL of distilled water. Uniform wheat seeds were selected and evenly placed in each dish, with 100 seeds per dish. Each treatment was replicated three times. Each treatment comprised three independent Petri dishes. Seeds were incubated for 7 days at 20 ± 0.5 °C and 70 ± 5% relative humidity under a 12-h photoperiod at 2,000 lx. Distilled water was replenished daily using a gravimetric method to maintain a consistent moisture level in the filter paper. Incubation conditions and watering schedules were identical across treatments, including CK. The number of germinated seeds was recorded at the same time each day. Germination was defined as the radicle reaching a length equal to that of the seed and the plumule reaching half the seed length. Germination potential and final germination percentage were calculated as the percentages of seeds germinated by days 4 and 7, respectively. Other germination indices were calculated from the daily counts. The calculation formulas were as follows (Ranalde Santana, 2006):


$$\text{GR}=(\frac{\text{Ng}}{\text{N}})\times 100\%$$

$$\text{GP}={\text{N}}_{\text{p}}/\text{N}\times 100$$

$$\text{GI}=\sum (\frac{\text{Gt}}{\text{Dt}})$$

VI=GI×S

$$\text{MGT}=\sum ({\text{G}}_{\text{t}}\times {\text{D}}_{\text{t}})/\sum {\text{G}}_{\text{t}}$$

$$\text{SVI}=\text{GR}\times {\text{L}}_{\text{s}}$$

$$\text{Z}=\sum [{\text{G}}_{\text{t}}({\text{G}}_{\text{t}}-1)/2]/[{\text{N}}_{\text{g}}({\text{N}}_{\text{g}}-1)/2]$$

$$\text{GRC}=\sum {\text{G}}_{\text{t}}/\sum ({\text{G}}_{\text{t}}\times {\text{D}}_{\text{t}})\times 100$$

(where N is the total number of tested seeds; Ng is the final number of normally germinated seeds; Np is the number of normally germinated seeds within the specified period; Gt is the number of newly germinated seeds on day t; Dt is the number of days after the start of the germination test; Ls is the mean seedling length; and Ws is the mean seedling fresh weight. GR denotes germination rate, GP denotes germination potential, GI denotes germination index, MGT denotes mean germination time, SVI denotes seedling vigor index, VI denotes vigor index, Z denotes germination synchronization index, and GRC denotes germination rate coefficient).

### 2.4 Field emergence dynamics, seedling establishment quality, and related evaluation indicators

From the fifth day after wheat sowing, the number of emerged seedlings in each 1 m^2^ area was recorded for each treatment at 1-day intervals. Plant height, root length, dry weight, and fresh weight were also measured. Emergence was complete by the fourteenth day, after which emergence rate, emergence dynamics, and field emergence rate-related indicators were determined. Wheat emergence dynamics were fitted using a logistic equation (Amorim et al., 2021; Martinez et al., 2017; Dias et al., 2011). where N_t_ is the number of emerged seedlings on day t after sowing; K is the theoretical maximum number of emerged seedlings; r is the emergence rate parameter; a is the initial position parameter; t is the number of days after sowing; and N_0_ is the total number of seeds sown. The variables t25, t50, t75, and t99 represent the time required for emergence to reach 25%, 50%, 75%, and 99%, respectively. The maximum emergence rate is denoted by Vmax, the mean emergence rate during the main emergence period by V25−75, the uniformity index by UI, and the emergence completion index by EUI. Nonlinear least−squares fitting was performed, and goodness of fit was evaluated using the coefficient of determination (R^2^).


$${\text{N}}_{\text{t}}=\frac{\text{K}}{1+{\text{e}}^{\text{a}-\text{rt}}}$$

$$\text{E}(\text{t})={\text{N}}_{\text{t}}/{\text{N}}_{0}\times 100$$

$${\text{t}}_{\text{p}}={\text{t}}_{\text{i}}+(\text{p}-{\text{E}}_{\text{i}})/(\text{E}(\text{i}+1)-{\text{E}}_{\text{i}})\times (\text{t}(\text{i}+1)-{\text{t}}_{\text{i}})$$

$${\text{tt}}_{\text{p}}={\text{t}}_{\text{i}}+\frac{\text{p}-{\text{E}}_{\text{i}}}{{\text{E}}_{\text{i}+1}-{\text{E}}_{\text{i}}}({\text{t}}_{\text{i}+1}-{\text{t}}_{\text{i}})$$

p=25, 50, 75, 99

$${\text{t}}_{25}={\text{t}}_{\text{p}}{|}_{\text{p}=25}{\text{t}}_{25}={\text{t}}_{\text{p}}{|}_{\text{p}=25}$$

$${\text{t}}_{50}={\text{t}}_{\text{p}}{|}_{\text{p}=50}$$

$${\text{t}}_{75}={\text{t}}_{\text{p}}{|}_{\text{p}=75}$$

$${\text{t}}_{99}={\text{t}}_{\text{p}}{|}_{\text{p}=99}$$

$$\text{Vmax}=\text{max}(\frac{{\text{E}}_{\text{i}+1}-{\text{E}}_{\text{i}}}{{\text{t}}_{\text{i}+1}-{\text{t}}_{\text{i}}})$$

$${\text{V}}_{25-75}=\frac{\text{E}({\text{t}}_{75})-\text{E}({\text{t}}_{25})}{{\text{t}}_{75}-{\text{t}}_{25}}$$

$$\text{MER}=\frac{\text{E}({\text{t}}_{99})}{{\text{t}}_{99}}$$

$$\text{UI}=\frac{1}{{\text{t}}_{75}-{\text{t}}_{25}}$$

$$\text{EUI}=\frac{{\text{N}}_{\text{f}}}{{\text{N}}_{0}}$$

### 2.5 Analysis of root architecture and root activity

Root observation devices were installed in the experimental field only during the second growing season (2025–2026) to facilitate root observation and recovery of intact root systems for scanning. Each device was cuboid, measured 30 × 10 × 45 cm, and had three wooden sides and one acrylic side. Each of the eight seed-dressing treatments was replicated three times under each sowing method, giving 48 devices in total. All devices were placed in the experimental field, with treatment combinations and replication matching the field split-plot experiment. Wheat was sown in the devices on the same day as in the field. Seeds were covered with approximately 5 cm of soil under soil-covered sowing and left uncovered on the soil surface under surface sowing, matching the corresponding field treatments. Root images were captured at 20, 40, and 60 days after sowing. Following the final imaging at 60 days after sowing, roots from 10 plants per device were collected at the jointing stage, washed, and scanned using an Epson Expression 10000XL scanner. The scanned images were analyzed using WinRHIZO to determine total root length, root volume, number of root forks, and root surface area. Root trait measurements were averaged across the 10 plants sampled within each device, and the resulting device-level means were reported in **Table 1** and used for statistical analysis. Each treatment combination comprised three independent device replicates (n=3). Root activity was measured separately using roots collected from 10 plants per field plot in both growing seasons (2024–2025 and 2025–2026). It was determined using the triphenyl tetrazolium chloride (TTC) method (Zhou et al., 2018).

**Table 1:** Effects of different seed-dressing treatments on root architecture and growth in wheat root observation devices in 2025–2026.

Year	S	T	TRL (cm)	TRSA (cm^2^)	TRV (cm^3^)	ARD (mm)	RTN	RFN
2025-2026	Surface sown	CK	195.3c	43.5c	0.64e	0.59b	223.3b	320.3e
		T_C_	378.1a	74.6a	1.16a	0.63ab	488.0a	924.1a
		T_B_	294.6b	57.6b	0.89bc	0.62ab	445.0a	694.5bc
		T_J_	294.8b	59.3b	0.95b	0.64ab	467.3a	730.6bc
		T_J+B_	298.4b	58.2b	0.94b	0.66ab	517.7a	648.7c
		T_C+B_	271.5b	54.6b	0.79cd	0.64ab	470.0a	829.2ab
		T_J+50%C_	222.3c	44.4c	0.74de	0.68a	365.3a	499.0d
		T_J+B+50%C_	360.6a	69.2a	1.10a	0.62ab	496.0a	736.4bc
		Mean	289.5a	57.6a	0.90a	0.63a	434.1a	672.9a
	Covered soil	CK	245.0d	47.6c	0.74d	0.52c	438.3bc	460.0d
		T_C_	402.9a	65.9a	0.86c	0.62ab	876.7a	856.1ab
		T_B_	256.7cd	48.0c	0.75cd	0.60b	365.0c	447.5d
		T_J_	305.8bc	64.4a	1.05a	0.65ab	376.7c	767.6b
		T_J+B_	259.1cd	55.0bc	1.00a	0.68a	437.3bc	527.4cd
		T_C+B_	322.9b	58.0ab	0.87bc	0.61ab	562.7b	627.2c
		T_J+50%C_	304.1bc	61.0ab	0.98ab	0.64ab	384.0c	546.3cd
		T_J+B+50%C_	326.7b	64.93a	0.99a	0.61ab	753.0a	933.7a
		Mean	302.9a	58.09a	0.91a	0.62a	524.2a	645.6a
	F-value	S	2.65*ns*	0.12*ns*	0.02*ns*	1.01*ns*	5.28*ns*	0.34*ns*
		T	16.97**	17.07**	19.27**	2.86*	14.52**	22.41**
		S×T	7.18**	8.095**	10.41**	2.25*ns*	6.416**	8.92**

The values in the table are mean ± standard error. Different lowercase letters for the same indicator indicate significant differences among treatments (P < 0.05). Asterisks above the values indicate significant differences among treatments: *, P < 0.05; **, P < 0.01; ***, P < 0.001; ns, not significant.

### 2.6 Investigation of stem and leaf growth and population quality

The uppermost fully expanded leaves from the main stem and tillers were sampled at the three-leaf stage (t_1_) and jointing stage (t_2_). For each sample, 0.1 g of fresh material was weighed and extracted in 10 mL of an acetone-ethanol mixture 1 : 1,v / v for 7 days. The absorbance of the extract was then measured at 645 nm and 663 nm, and chlorophyll content was calculated. The remaining leaves and stems were deactivated at 105 °C for 30 min, then oven-dried at 85 °C to constant weight and weighed. These measurements were used to calculate leaf area index, leaf area ratio at the jointing stage, and crop growth rate, relative growth rate, growth function (G value), and seedling vigor index from the three-leaf stage to the jointing stage.


$$\text{Total chlorophyll }(\text{mg }{\text{g}}^{-1})=\frac{(20.2A_{645}+8.02A_{663})V}{1000w}$$

$$\text{LWR}=\frac{LW}{W}$$

$$\text{LAI}=\frac{L_{2}}{S}$$

$$\text{LAR}=\frac{L_{2}}{W}$$

$$\text{RGR }(\text{mg }{\text{g}}^{-1}\;{\text{d}}^{-1})=\frac{\ln W_{2}-\ln W_{1}}{t_{2}-t_{1}}$$

$$\text{CGR }(\text{g }{\text{m}}^{-2}\;{\text{d}}^{-1})=\frac{W_{2}-W_{1}}{(t_{2}-t_{1})S}$$

$$\text{G}=\frac{W_{2}-W_{1}}{t_{2}-t_{1}}$$

where V is the volume of the chlorophyll extract; w is the fresh weight of the material; W, L, and L_W_ are plant dry weight, leaf area, and leaf dry weight, respectively; W_1_, W_2_, L_1_, and L_2_ are plant dry weight and leaf area at t_1_ and t_2_, respectively; and S is the land area.

### 2.7 Determination of yield and yield components

At maturity, wheat plants were harvested from a 1 m^2^ area in each plot. The harvested plants were threshed, sun-dried, and weighed, and thousand-grain weight was determined. Grain moisture content was measured using a grain moisture meter, and grain weight was adjusted to a moisture content of 13.0% after drying, as follows:


$$W_{\text{actual}}=W_{\text{measured}}\times \frac{1-M}{1-0.13}$$

For each treatment, three representative 1 m^2^ sampling areas were also selected to count effective spikes. In addition, 30 spikes were randomly sampled and threshed to determine the number of grains per spike. In the equation above, *M* denotes the measured grain moisture content expressed as a decimal.

### 2.8 Data processing

Data were organized in Microsoft Excel 2021 and analyzed using IBM SPSS Statistics 26.0 and GraphPad Prism 10.5. Field and root data were analyzed by growing season using a linear mixed model for a split-plot design with three blocks. Sowing method, seed-dressing treatment, and their interaction were fixed effects; block and block × sowing method were random effects. Sowing method was tested against the whole-plot error, and seed-dressing treatment and their interaction against the subplot residual error. Germination data were analyzed using one-way ANOVA with three independent Petri dishes per treatment. Residual normality and variance homogeneity were assessed using Shapiro–Wilk and Levene tests, respectively. Means were compared using Duncan’s multiple range test (P < 0.05), with simple effects examined for significant interactions. Different lowercase letters indicate significant differences within each comparison group. Data are presented as means ± SE of three independent subplots, root observation devices, or Petri dishes, as appropriate. Figures were generated using GraphPad Prism 10.5 and ggplot2 in R 4.5.3.

## 3 Results

### 3.1 Effects of seed-dressing treatments on wheat seed germination and seed vigor

**Table 2** shows that seed-dressing treatment significantly affected wheat seed germination and early seedling vigor in both growing seasons. In 2024–2025, T_C_ had the highest GR and SVI, exceeding CK by 12.20% and 47.69%, respectively. T_C_, T_B_, and T_C+B_ also had higher germination potential than CK. T_J+B_ had the highest numerical GI, while T_J_ had the highest numerical VI. These values were 27.94% and 53.64% higher than those of CK, respectively. However, T_C_ did not significantly increase GI or VI relative to CK. T_J+50%C_ reduced GR to 81.33%, compared with 84.67% under CK. T_C+B_ had the shortest numerical MGT, but it did not differ significantly from CK. In 2025–2026, Ju Chuan + brassinolide + 50% Coolas (T_J+B+50%C_) achieved the highest numerical germination rate (GR), exceeding that of CK by 12.89%, and increased the germination index (GI) and seedling vigor index (SVI) by 96.12% and 22.88%, respectively. Coolas (T_C_) produced the highest vigor index (VI), representing a 97.69% increase relative to CK. Ju Chuan + brassinolide (T_J+B_) also increased GI, SVI, and VI by 92.14%, 20.25%, and 93.08%, respectively. In addition, T_J+B_ reduced the mean germination time (MGT) to 3.73 d, which was 9.25% shorter than that of CK. T_J+B+50%C_ did not differ significantly from T_C_ or T_J+B_ in GR or SVI. T_C_ did not differ significantly from T_J+B_, T_J+50%C_, or T_J+B+50%C_ in VI.

**Table 2:** Comprehensive evaluation of wheat seed germination traits and vigor in 2024–2026.

Year	T	GR/(%)	GP/(%)	GI	MGT/(d)	SVI	VI	Z	GRC
2024-2025	CK	84.67d	62.33b	16.39cd	4.32bcd	969.12d	108.73c	0.90b	0.23ab
	TC	95.00a	75.67a	14.58d	4.31bcd	1431.25a	131.60bc	0.94a	0.22b
	TB	90.00bc	70.67a	19.61ab	4.25cd	1229.33b	155.39ab	0.90b	0.24ab
	TJ	90.33bc	58.33b	19.64ab	4.72ab	1167.21b	167.05a	0.89b	0.21cd
	TJ+B	89.67bc	60.33b	20.97a	5.00a	1097.24bc	165.34a	0.85c	0.19d
	TC+B	91.33b	73.67a	19.99ab	4.01d	1193.33b	156.38ab	0.89b	0.23a
	TJ+50%C	81.33e	61.00b	17.34bc	4.37bcd	1019.22cd	144.44ab	0.90b	0.24b
	TJ+B+50%C	88.33c	62.00b	19.21ab	4.46bc	1134.81bc	165.27a	0.89b	0.21bc
	F-value	27.81**	12.11**	7.06**	5.89**	12.47**	5.95**	4.39**	6.46**
2025-2026	CK	82.67c	59.33c	19.08e	4.11bc	1376.98b	151.69d	0.85a	0.24abc
	T_C_	92.00a	67.67ab	32.33bc	4.21abc	1676.31a	299.88a	0.73bc	0.24bcd
	T_B_	88.00ab	59.67c	27.81d	4.45ab	1491.00ab	211.84c	0.81ab	0.23cd
	T_J_	83.67bc	60.33c	30.16cd	3.83c	1320.42b	232.07bc	0.70bc	0.26ab
	T_J+B_	90.33a	67.33ab	36.66a	3.73c	1655.95a	292.88a	0.54d	0.27a
	T_C+B_	88.33ab	63.67bc	30.39cd	4.69a	1547.94ab	247.98b	0.79ab	0.21d
	T_J+50%C_	89.67a	73.33a	34.31ab	4.59ab	1557.85ab	285.83a	0.66c	0.22cd
	T_J+B+50%C_	93.33a	71.67a	37.42a	4.25abc	1692.06a	285.81a	0.68c	0.24bcd
	F-value	5.39**	6.24**	28.50**	4.41**	3.64*	23.06**	8.72**	5.03**

Different lowercase letters within the same indicator denote significant differences among treatments (P < 0.05). Asterisks indicate significance levels: *P < 0.05, **P < 0.01, and ***P < 0.001.

### 3.2 Effects of sowing method and seed-dressing treatment on wheat emergence and seedling establishment

Emergence dynamics differed between sowing methods in both years, with treatment differences being more pronounced under uncovered sowing (**Figure 2A**). Soil-covered sowing produced earlier and more synchronized emergence, with generally higher final seedling numbers than uncovered sowing during the observation period. In 2025–2026, Sowing method, seed-dressing treatment, and their interaction significantly affected most emergence parameters (**Table 3**). The variables t25, t50, t75, and t99 represent the time required for emergence to reach 25%, 50%, 75%, and 99%, respectively. The maximum emergence rate is denoted by Vmax, the mean emergence rate during the early emergence period by V25–75, the mean emergence rate by MER, the uniformity index by UI, and the emergence completion index by EUI. Soil covering reduced t25, t50, t75, and t99 from 3.08, 5.48, 7.71, and 15.08 d to 2.39, 3.84, 5.27, and 9.41 d, respectively. It also increased Vmax, V25–75, and MER from 9.39, 7.02, and 7.66% d^–1^ to 16.02, 12.00, and 12.47% d^–1^, respectively. Under uncovered sowing, T_B_ produced the greatest acceleration of emergence. Relative to CK, TB reduced t50 and t99 by 43.7% and 45.2%, respectively, and increased numerical MER by 87.9%. Under soil-covered sowing, T_B_ produced the highest Vmax (24.72% d^–1^), whereas T_J+B_ produced the highest MER (14.13% d^–1^). By contrast, Ju Chuan alone (T_J_) and Ju Chuan plus half-rate Coolas (T_J+50%C_) did not outperform CK in V25–75 or MER under soil-covered sowing.

**Figure 2:**
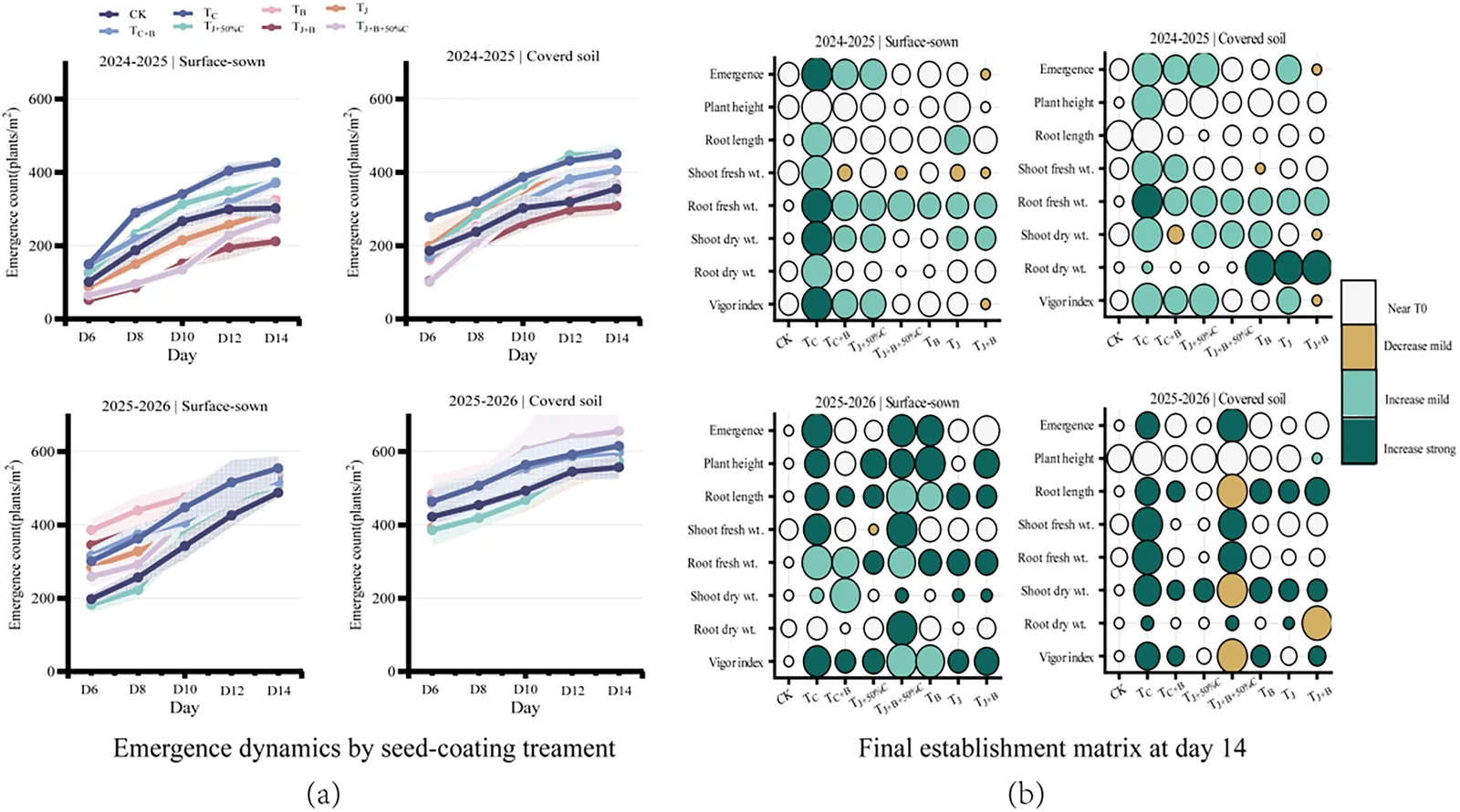
Field emergence dynamics and morphological establishment from 6 to 14 days after sowing during 2024-2026. **(A)** Seedling emergence dynamics over the first 14 days. **(B)** Seedling morphogenesis on day 14.

**Table 3:** Effects of different seed-dressing treatments on field emergence indicators in 2025-2026.

S	T	t50/(d)	t25/(d)	t75/(d)	t99/(d)	Vmax /(%·d^-1^)	V25-75/(%·d^-1^)	MER/(%·d^-1^)	UI	EUI
Surface sown	CK	6.75 ± 0.68ab	3.98 ± 0.38b	9.02 ± 0.47ab	17.69 ± 1.09a	7.02 ± 0.68c	5.11 ± 0.5d	5.78 ± 0.49d	1.34 ± 0.01de	0.55 ± 0.01ab
	T_C_	5.26 ± 0.47de	2.53 ± 0.15d	7.05 ± 0.4c	14.57 ± 1.16b	9.31 ± 0.74bc	6.78 ± 0.54c	7.93 ± 0.29bc	1.71 ± 0.09b	0.53 ± 0.02b
	T_C+B_	4.57 ± 0.5ef	2.48 ± 0.22d	6.66 ± 0.81c	13.3 ± 1.79b	9.32 ± 0.99bc	6.79 ± 0.72c	8.08 ± 0.69bc	1.49 ± 0.03c	0.52 ± 0.01b
	T_J+50%C_	7.18 ± 0.3a	4.29 ± 0.28a	9.74 ± 0.23a	17.9 ± 1.37a	8.85 ± 0.34bc	6.63 ± 0.23c	6.11 ± 0.47d	1.27 ± 0.06e	0.58 ± 0.04a
	T_J+B+50%C_	6.32 ± 0.68bc	3.29 ± 0.12c	9.11 ± 0.87ab	18.01 ± 1.48a	7.8 ± 0.34c	5.68 ± 0.25cd	6.68 ± 0.38cd	1.68 ± 0.07b	0.53 ± 0.01b
	T_B_	3.8 ± 0.13g	2.39 ± 0.09d	5.21 ± 0.22d	9.7 ± 0.56c	14.06 ± 1.81a	10.57 ± 0.74a	10.86 ± 1.07a	1.42 ± 0.03cd	0.57 ± 0.01ab
	T_J_	5.66 ± 0.43cd	3.19 ± 0.13c	8.47 ± 0.39b	17.02 ± 0.96a	7.68 ± 0.82c	6.5 ± 0.63c	6.66 ± 0.54cd	1.85 ± 0.07a	0.52 ± 0.01b
	T_J+B_	4.28 ± 0.51fg	2.48 ± 0.22d	6.41 ± 0.33c	12.48 ± 0.74b	11.09 ± 1.18b	8.08 ± 0.86b	9.19 ± 0.67b	1.64 ± 0.08b	0.54 ± 0.01ab
	Mean	5.48 ± 0.46a	3.08 ± 0.20a	7.71 ± 0.46a	15.08 ± 1.15a	9.39 ± 0.86b	7.02 ± 0.56b	7.66 ± 0.58a	1.55 ± 0.06a	0.54 ± 0.02b
Covered soil	CK	4.63 ± 0.26a	2.33 ± 0.09a	5.73 ± 0.21ab	9.41 ± 1.31abc	12.03 ± 0.75d	12.34 ± 0.82bc	11.97 ± 1.14bc	1.29 ± 0.04c	0.59 ± 0.04ab
	T_C_	3.66 ± 0.22b	2.38 ± 0.02a	4.94 ± 0.44bc	9.01 ± 1.15bc	17.35 ± 2.32bc	12.79 ± 0.7bc	12.62 ± 0.42ab	1.66 ± 0.05ab	0.59 ± 0.03ab
	T_C+B_	3.56 ± 0.14b	2.38 ± 0.02a	4.73 ± 0.27c	8.46 ± 0.66c	18.89 ± 2.21b	13.09 ± 0.45b	13.62 ± 1.27a	1.55 ± 0.04b	0.6 ± 0.01a
	T_J+50%C_	3.86 ± 0.33b	2.45 ± 0.2a	5.35 ± 0.52bc	11.14 ± 1.45ab	11.67 ± 0.39d	8.99 ± 0.46d	11.17 ± 0.75c	1.57 ± 0.07b	0.54 ± 0.04b
	T_J+B+50%C_	3.85 ± 0.35b	2.51 ± 0.18a	5.77 ± 0.2ab	9.06 ± 0.99bc	15.97 ± 0.4c	11.69 ± 0.39c	12.95 ± 0.8ab	1.63 ± 0.06b	0.58 ± 0.03ab
	T_B_	3.56 ± 0.17b	2.47 ± 0.22a	4.65 ± 0.44c	7.79 ± 0.83c	24.72 ± 2.39a	16.24 ± 0.91a	13.65 ± 1.01a	1.75 ± 0a	0.62 ± 0.05a
	T_J_	4.11 ± 0.19ab	2.28 ± 0.07a	6.35 ± 0.41a	11.44 ± 1.31a	11.5 ± 0.21d	8.1 ± 0.46d	9.67 ± 0.6d	1.57 ± 0.08b	0.54 ± 0.03b
	T_J+B_	3.48 ± 0.2b	2.32 ± 0.05a	4.64 ± 0.45c	9 ± 0.23bc	16.05 ± 1.77c	12.78 ± 0.89bc	14.13 ± 1.13a	1.62 ± 0.06b	0.6 ± 0.04a
	Mean	3.84 ± 0.23b	2.39 ± 0.11b	5.27 ± 0.37b	9.41 ± 0.99b	16.02 ± 1.31a	12.00 ± 0.64a	12.47 ± 0.89a	1.44 ± 0.05b	0.58 ± 0.03a
F-value	S	108.17**	88.63*	44.02**	286.26**	526.28**	123.93**	63.00**	3.79ns	46.76*
	T	19.96**	26.45**	29.96**	17.12**	39.75**	49.76**	19.25**	32.350**	2.53*
	S×T	8.47**	25.99**	9.29**	5.66**	7.64**	13.59**	3.818**	17.312**	2.43*

The values in the table are mean ± standard error, and different lowercase letters for the same indicator indicate significant differences among treatments (p < 0.05). Asterisks above the values indicate significant differences among treatments: *, P < 0.05; **, P < 0.01; ***, P < 0.001. The same applies below.

The day-14 assessments showed that seed-dressing effects on seedling morphology varied among traits and sowing conditions (**Figures 2A, B**). T_C_ consistently increased emerged seedling number, root fresh weight, shoot dry weight, and seedling vigor index under both sowing methods in both years. T_B_ mainly increased root length, root dry weight, and seedling vigor index. Under soil-covered sowing in 2025–2026, T_J+B+50%C_ increased emergence, root length, shoot fresh weight, root fresh weight, Soil covering increased plant height and shoot biomass in both years, whereas root length and root biomass responses differed between years (**Figure 3**). Root length was lower under soil-covered sowing in 2024–2025, but higher in 2025–2026. Root fresh weight did not differ significantly between sowing methods in 2024–2025. Root dry weight did not differ significantly between sowing methods in 2025–2026.

**Figure 3:**
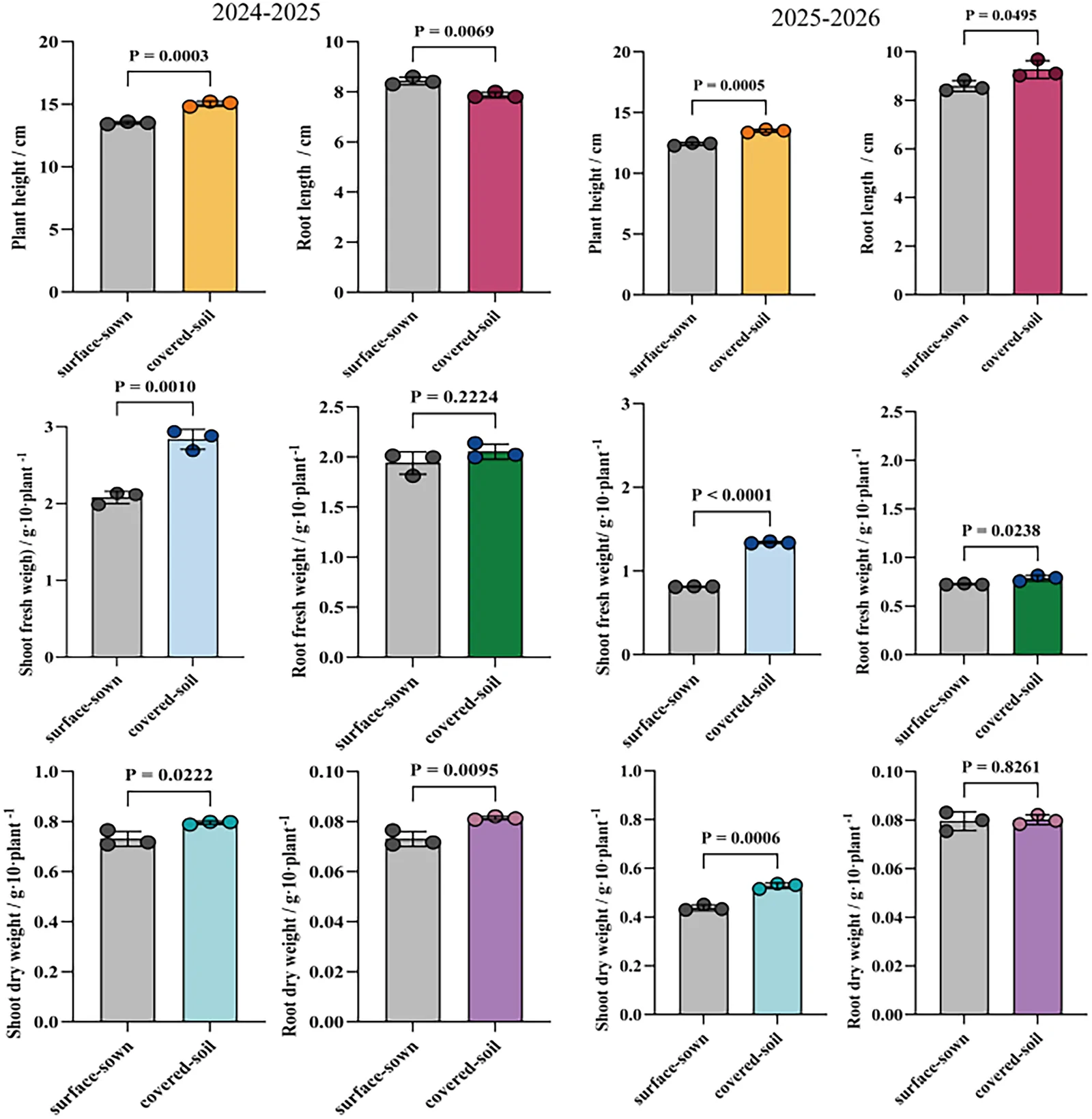
Plant height, root length, and shoot and root fresh and dry weights at 14 days after sowing under uncovered surface sowing and soil-covered sowing in 2024–2025 and 2025–2026. Comparisons summarize the seed-dressing treatments within each growing season. P-values are shown above the corresponding comparisons.

### 3.3 Effects of sowing method and seed-dressing treatment on wheat seedling growth and chlorophyll content

In 2024–2025, sowing method, seed-dressing treatment, and their interaction affected seedling growth indices to varying degrees (**Table 4**). Sowing method significantly affected leaf area ratio (LAR), crop growth rate (CGR), growth function (G), relative growth rate (RGR), and leaf weight ratio, but not leaf area index (LAI). Compared with uncovered sowing, soil covering reduced mean LAR from 83.2 to 75.9 cm^2^ g^–1^ and increased CGR from 11.68 to 12.67 g m^-2^ d^–1^. The G value and RGR also increased from 0.117 to 0.127 and from 0.038 to 0.040, respectively. T_C_ showed the strongest overall growth response under both sowing methods. Under uncovered sowing, T_C_ produced a CGR of 13.35, a G value of 0.134, an RGR of 0.042, and an LAI of 1.47. Under soil-covered sowing, T_C_ produced the highest CGR and G values, reaching 14.33 and 0.143, respectively. T_J+50%C_ and T_J+B+50%C_ also showed relatively high growth indices under soil-covered sowing. For T_J+B+50%C_, CGR, G, and RGR reached 13.98, 0.140, and 0.043, respectively. By contrast, T_J+B_ had comparatively low CGR and G values among the seed-dressing treatments under both sowing methods. In 2025–2026, sowing method significantly affected LAR, CGR, G value, RGR, and LWR, but not LAI (**Table 5**). Seed-dressing treatment and its interaction with sowing method significantly affected all six indices. Under uncovered sowing, T_J+B_ had the highest numerical CGR, G value, RGR, and LAI. Its CGR was 39.32% higher than that of CK and did not differ significantly from T_J_. T_C+B_ and T_J+B+50%C_ did not differ significantly from CK in CGR, G value, or RGR. Under soil-covered sowing in 2025–2026, T_C_ had the highest CGR, G value, and RGR. Its CGR exceeded CK by 71.16%. TJ_+B+50%C_ had the second-highest numerical CGR. T_C_ also had the highest numerical LAI, but it did not differ significantly from T_J+50%C_ or T_J+B+50%C_.

**Table 4:** Effects of different seed-dressing treatments on leaf area and growth dynamics at the wheat seedling stage in 2024-2025.

Year	S	T	LAR (cm^2^·g^-1^)	CGR g·(m^2^·d)^-1^	G value (g·d^-1^)	RGR g·(g·d)^-1^	LWR	LAI
2024-2025	Surface sown	CK	87.1ab	9.92f	0.099f	0.034g	0.467d	0.97b
		T_C_	99.5a	13.35a	0.134a	0.042a	0.505b	1.47a
		T_C+B_	79.4b	12.78b	0.128b	0.042ab	0.519a	1.16b
		T_J+50%C_	83.3ab	12.36c	0.124bc	0.039bc	0.492bc	1.17b
		T_J+B+50%C_	75.6b	11.48d	0.115d	0.039de	0.488c	0.96b
		T_B_	77.6b	11.17d	0.112d	0.037e	0.492c	0.96b
		T_J_	76.9b	11.98c	0.120c	0.039cd	0.520a	1.01b
		T_J+B_	85.4ab	10.40e	0.104e	0.036f	0.499bc	0.98b
		Mean	83.2a	11.68b	0.117b	0.038b	0.498a	1.093a
	Covered soil	CK	79.1a	10.43g	0.104g	0.036e	0.497b	0.94b
		T_C_	73.3a	14.33a	0.143a	0.044a	0.472c	1.23ab
		T_C+B_	74.2a	11.58e	0.116e	0.038d	0.498b	1.01ab
		T_J+50%C_	83.3a	13.72bc	0.137bc	0.042b	0.499b	1.31a
		T_J+B+50%C_	73.3a	13.98ab	0.140ab	0.043ab	0.495b	1.14ab
		T_B_	80.3a	13.48c	0.135c	0.042b	0.524a	1.18ab
		T_J_	72.6a	12.73d	0.127d	0.041c	0.467c	1.05ab
		T_J+B_	77.2a	11.11f	0.111f	0.037d	0.503b	0.99b
		Mean	75.9b	12.67a	0.127a	0.040a	0.494b	1.102a
	F-value	S	56.81*	293.12**	315.59**	675.00**	70.33*	1.21^ns^
		T	1.39ns	133.25**	128.00**	109.30**	10.21**	6.71**
		S×T	1.272ns	31.470**	29.521**	21.624**	26.111**	2.406*

Different lowercase letters for the same indicator indicate significant differences among treatments (P < 0.05). Asterisks above the values indicate significant differences among treatments: *, P < 0.05; **, P < 0.01; ***, P < 0.001; ns, not significant.

**Table 5:** Effects of different seed-dressing treatments on leaf area and growth dynamics at the wheat seedling stage in 2025-2026.

Year	S	T	LAR (cm^2^·g^-1^)	CGR g·(m^2^·d)^-1^	G value (g·d^-1^)	RGR g·(g·d)^-1^	LWR	LAI
2025-2026	Surface sown	CK	107.21a	9.64de	0.096de	0.034d	0.519c	1.15bc
		T_C_	102.07ab	10.93cd	0.109cd	0.037c	0.568a	1.31ab
		T_C+B_	90.89bc	9.57de	0.096de	0.034d	0.568a	1.02cd
		T_J+50%C_	104.66ab	11.78bc	0.118bc	0.039bc	0.561ab	1.39ab
		T_J+B+50%C_	85.02c	9.17e	0.092e	0.032d	0.512c	0.90d
		T_B_	94.49abc	11.01cd	0.110cd	0.037c	0.577a	1.17bc
		T_J_	87.65c	12.55ab	0.125ab	0.040ab	0.550abc	1.21abc
		T_J+B_	96.69abc	13.43a	0.134a	0.042a	0.526bc	1.41a
		Mean	96.09a	11.01b	0.110b	0.037b	0.547a	1.20a
	Covered soil	CK	87.65a	10.23e	0.102e	0.035e	0.525b	1.02cd
		T_C_	77.01ab	17.51a	0.175a	0.049a	0.483c	1.53a
		T_C+B_	90.27a	10.96de	0.110de	0.037de	0.572a	1.17bc
		T_J+50%C_	89.37a	12.98bc	0.130bc	0.041bc	0.479c	1.32ab
		T_J+B+50%C_	84.55a	14.23b	0.142b	0.043b	0.532b	1.37ab
		T_B_	66.63b	11.75cd	0.118cd	0.039cd	0.440d	0.89d
		T_J_	79.93ab	13.01bc	0.130bc	0.041bc	0.528b	1.19bc
		T_J+B_	81.99a	12.78bc	0.128bc	0.041bc	0.506bc	1.18bc
		Mean	82.18b	12.93a	0.129a	0.041a	0.508b	1.21a
	F-value	S	470.81**	160.06**	149.73**	142.10**	140.29**	1.25ns
		T	3.66**	18.01**	17.97**	19.80**	4.21**	7.64**
		S×T	2.70*	13.69**	13.74**	12.68**	8.85**	5.96**

Different lowercase letters for the same indicator indicate significant differences among treatments (P < 0.05). Asterisks above the values indicate significant differences among treatments: *, P < 0.05; **, P < 0.01; ***, P < 0.001; ns, not significant.

Chlorophyll content differed among seed-dressing treatments, but treatment rankings were generally consistent across years and sowing methods (**Figure 4**). In 2024–2025, T_C_ produced the highest chlorophyll content under both sowing methods and significantly exceeded CK. Under uncovered sowing, T_C+B_ ranked second, while T_B_, T_J+50%C_, and T_J+B+50%C_ also exceeded CK to varying degrees. Chlorophyll content was generally lower in 2025–2026, although treatment differences remained evident. T_C_ and T_J+B+50%C_ maintained relatively high chlorophyll contents under both sowing methods. Under uncovered sowing in 2025–2026, T_B_ and T_J_ did not differ significantly from CK. Under soil-covered sowing, all seed-dressing treatments exceeded CK.

**Figure 4:**
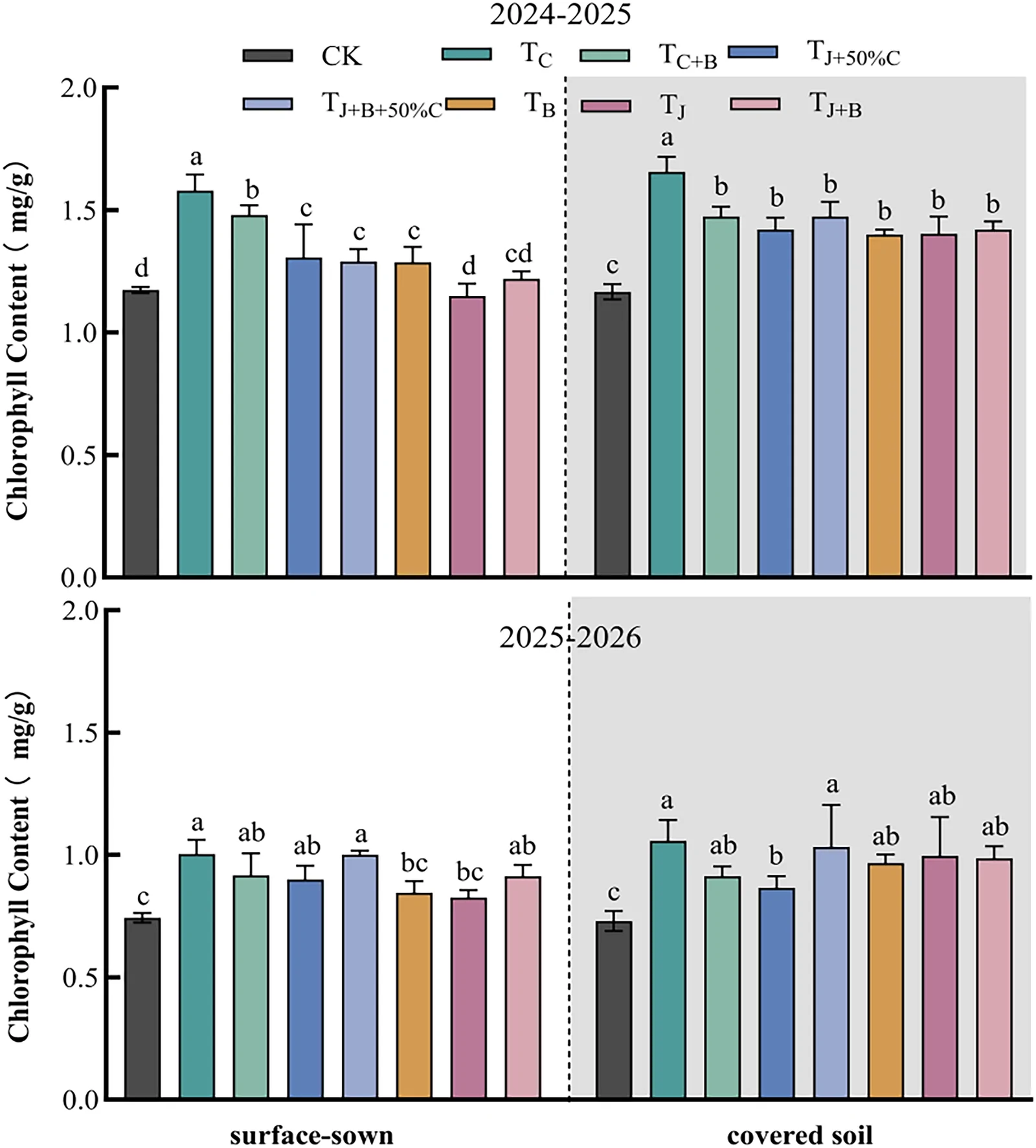
Effects of different seed-dressing treatments on chlorophyll content at the seedling stage during 2024-2026.

### 3.4 Effects of sowing method and seed-dressing treatment on wheat root activity and architecture

Field wheat root vitality differed among seed-dressing treatments in both growing seasons (**Figure 5**). In 2024–2025, T_C_ had the highest root activity under both sowing methods and significantly exceeded all other treatments. Under uncovered surface sowing, T_C+B_ did not differ significantly from CK, whereas the remaining seed-dressing treatments had higher root activity. Under soil-covered sowing, all seed-dressing treatments had higher root activity than CK. In 2025–2026, root activity also varied among treatments, with treatment rankings differing between sowing methods (**Figure 5B**). Under uncovered sowing, T_J+B+50%C_ and T_B_ produced the highest numerical root activity, but neither differed significantly from T_C_. Under soil-covered sowing, T_C_ and T_J+B+50%C_ produced the highest values.

**Figure 5:**
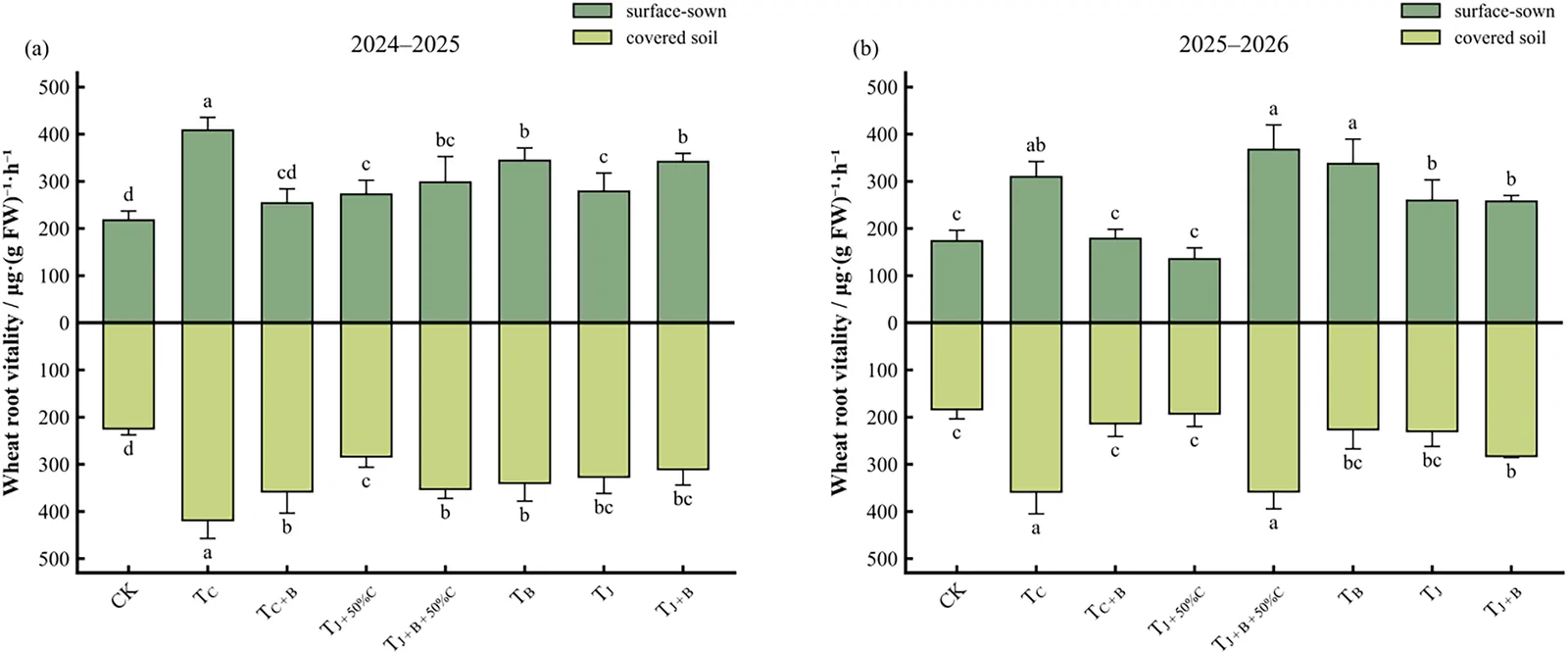
Effects of different seed-dressing treatments on root activity under field conditions during the 2024–2026 growing seasons. Bars above and below the central axis represent surface sowing and soil-covered sowing, respectively; all root activity values are positive. Different lowercase letters indicate significant differences among treatments within each sowing method and growing season (P < 0.05).

In 2025–2026, seed-dressing treatment significantly affected root activity and all measured root morphological traits. These traits included total root length (TRL), total root surface area (TRSA), total root volume (TRV), average root diameter (ARD), root tip number (RTN), and root fork number (RFN). Sowing method had no significant main effect on root morphology, whereas its interaction with seed-dressing treatment significantly affected all traits except ARD. Root activity also varied among treatments, with treatment rankings differing between sowing methods (**Figure 5**). Under uncovered sowing, T_J+B+50%C_ and T_B_ produced the highest root activity, followed by T_C_. Under soil-covered sowing, T_C_ and T_J+B+50%C_ produced the highest values.

Under uncovered sowing, T_C_ increased TRL, TRSA, and TRV by 93.6%, 71.5%, and 81.2%, respectively, relative to CK (**Table 1**). It also increased RTN and RFN by 118.5% and 188.5%, respectively. T_J+B+50%C_ increased TRL by 84.6% and increased RTN and RFN by 122.1% and 129.9%, respectively. T_J+50%C_ produced a larger ARD but smaller changes in TRL and TRSA. Under soil-covered sowing, T_C_ increased TRL by 64.4%, RTN by approximately 100%, and RFN by 86.1%. T_J+B+50%C_ produced the highest RFN, exceeding CK by 103.0%, and increased RTN by 71.8%. T_J_ increased TRV and ARD, but its RTN remained below that of CK. Representative root images agreed with the quantitative measurements, showing denser and more extensively branched root systems under T_C_ and T_J+B+50%C_ than under CK (**Figure 6**).

**Figure 6:**
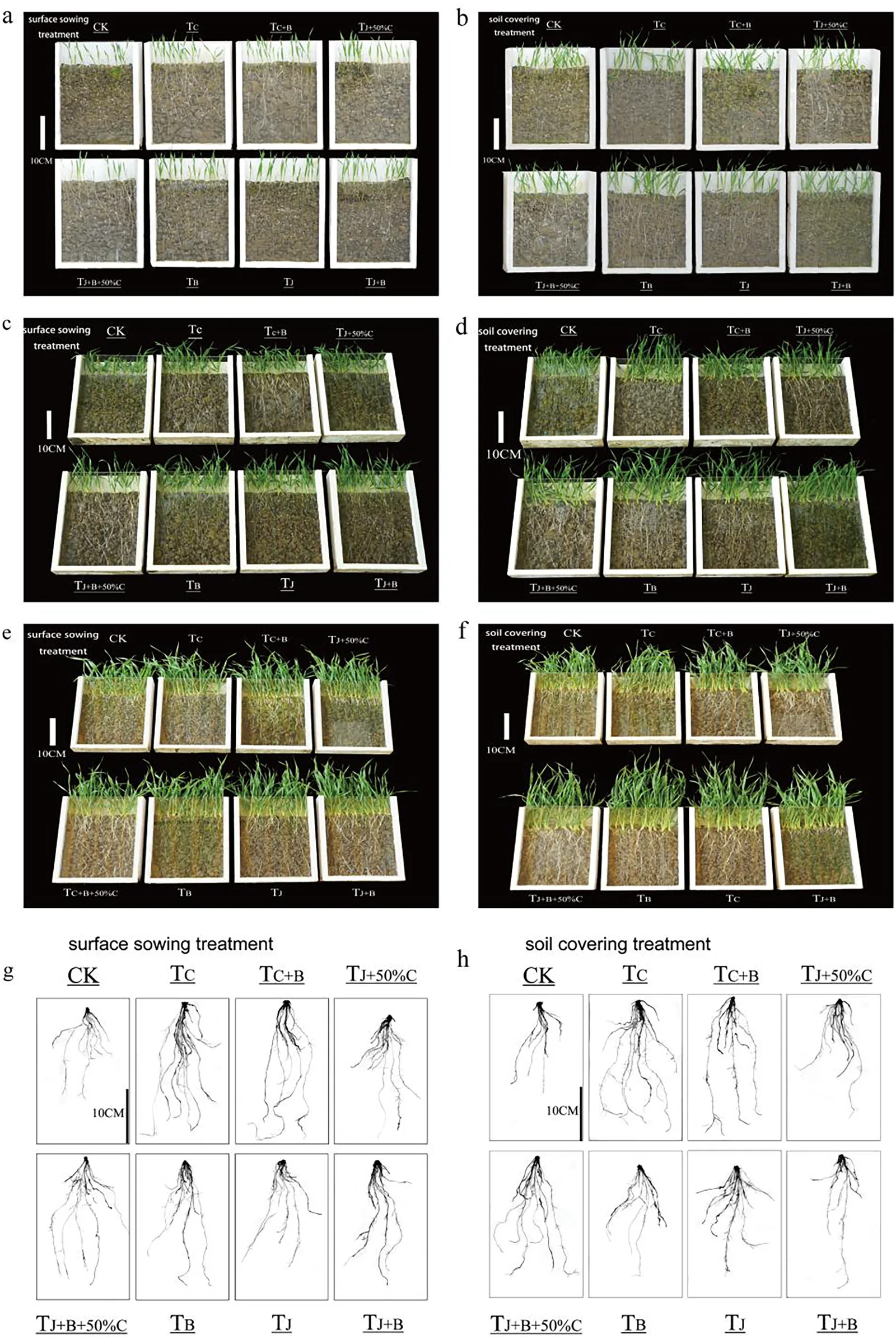
Effects of different seed-dressing treatments on aboveground and belowground growth phenotypes in root observation devices in 2025-2026. **(A, B)** Shoot and root phenotypes at 20 days. **(C, D)** Shoot and root phenotypes at 40 days. **(E, F)** Shoot and root phenotypes at 60 days. **(G, H)** Scanned root images at 60 days. Panels A, C, E, and g show the uncovered sowing method, whereas panels B, D, F, and h show the soil-covered sowing method. Panels G and H present scanned root images.

### 3.5 Effects of sowing method and seed-dressing treatment on wheat grain yield and yield components

Sowing method and seed-dressing treatment significantly affected grain yield and most yield components, with interaction effects varying between years (**Tables 6**, **7**). In 2024–2025, sowing method affected yield, grains per spike, and thousand-grain weight. Seed-dressing treatment and its interaction with sowing method affected all four measured traits. In 2025–2026, sowing method affected yield, effective spike number, and grains per spike, but not thousand-grain weight. Seed-dressing treatment affected all four traits, whereas the interaction was not significant for thousand-grain weight.

**Table 6:** Effects of different seed-dressing treatments on wheat yield under uncovered and soil-covered sowing in 2024-2025.

S	T	Grain yield (kg ha^-1^)	Fertile spikes (×10^4^ ha^-1^)	Grain number per spike	Thousand grain weight (g)
Surface sown	CK	6124.5 ± 191.0e	516.0 ± 7.4c	34.7 ± 0.3e	41.7 ± 0.9d
	T_C_	7324.4 ± 95.7a	572.9 ± 2.0a	37.3 ± 0.5bcd	44.1 ± 0.9ab
	T_B_	6482.7 ± 133.6de	522.0 ± 11.5c	38.3 ± 0.4ab	42.2 ± 1.1cd
	T_J_	6771.8 ± 170.2bc	524.2 ± 6.5c	38.9 ± 0.9a	45.1 ± 0.8a
	T_J+B_	6301.6 ± 160.3e	548.4 ± 9.0b	37.9 ± 0.4abc	39.9 ± 1.1e
	T_C+B_	6729.1 ± 149.9bc	567.7 ± 8.5a	36.7 ± 0.6cd	43.4 ± 1.2bc
	T_J+50%C_	6924.5 ± 48.3b	551.7 ± 8.1b	36.4 ± 0.7d	44.4 ± 0.5ab
	T_J+B+50%C_	6677.0 ± 193.6cd	527.0 ± 9.6c	36.7 ± 0.6cd	44.3 ± 0.6ab
	Mean	6666.96 ± 142.8b	541.2 ± 7.8b	37.1 ± 0.6b	43.1± 0.9b
Covered soil	CK	6593.7 ± 161.7d	546.4 ± 5.5c	40.4 ± 1.1c	42.9 ± 1.1b
	T_C_	7418.1 ± 168.0a	589.5 ± 15.7a	43.7 ± 0.7a	45.6 ± 0.3a
	T_B_	7088.7 ± 93.6b	537.9 ± 11.1d	41.2 ± 0.8bc	44.8 ± 1.0a
	T_J_	6881.9 ± 178.7bc	513.7 ± 10.8e	42.4 ± 0.5b	45.3 ± 0.9a
	T_J+B_	7098.1 ± 106.6b	532.4 ± 14.2cd	42.1 ± 1.1b	45.4 ± 0.4a
	T_C+B_	6949.8 ± 125.7bcd	544.2 ± 10.1c	41.9 ± 0.9b	45.3 ± 0.5a
	T_J+50%C_	6862.9 ± 196.3bc	584.4 ± 8.1a	41.2 ± 0.5bc	45.3 ± 0.8a
	T_J+B+50%C_	6714.6 ± 147.3cd	562.0 ± 5.6b	41.1 ± 0.7bc	44.4 ± 1.1a
	Mean	6950.99 ± 147.2a	551.3 ± 10.1a	41.8 ± 0.8a	44.9 ± 0.8a
F-value	S	104.15**	4.06ns	173.14**	28.09*
	T	21.61**	36.32**	11.27**	10.62**
	S×T	6.36**	11.18**	3.60**	6.53**

Different lowercase letters for the same indicator indicate significant differences among treatments (P < 0.05). Asterisks above the values indicate significant differences among treatments: *, P < 0.05; **, P < 0.01; ***, P < 0.001; ns, not significant.

**Table 7:** Effects of different seed-dressing treatments on wheat yield under uncovered and soil-covered sowing in 2025-2026.

S	T	Grain yield (kg ha^-1^)	Fertile spikes (×10^4^ ha^-1^)	Grain number per spike	Thousand grain weight (g)
Surface sown	CK	6012.4 ± 165.3e	420.7 ± 11.7d	37.4 ± 0.9bc	46.8 ± 0.2c
	T_C_	7294.7 ± 231.7a	503.3 ± 10.6b	38.8 ± 1ab	48.3 ± 1.3abc
	T_B_	6728.5 ± 158.9bc	538.3 ± 12.7a	35.5 ± 0.3d	47.2 ± 0.6bc
	T_J_	6841.4 ± 185.2b	475.7 ± 14.6c	38.6 ± 0.4b	47.6 ± 1.1abc
	T_J+B_	6400.5 ± 153.7d	502.7 ± 4.2b	38.1 ± 0.7b	48.6 ± 0.7ab
	T_C+B_	6424.8 ± 147.9cd	525.3 ± 10.2a	38 ± 0.3b	49 ± 0.8a
	T_J+50%C_	6529.5 ± 178.2cd	489.7 ± 10.6bc	36.3 ± 0.4cd	46.9 ± 0.6c
	T_J+B+50%C_	7323.8 ± 226.8a	487.3 ± 14.6bc	40 ± 0.3a	47.8 ± 0.4abc
	Mean	6694.4 ± 181.0b	492.9 ± 11.1b	37.8 ± 0.1b	47.8 ± 0.2a
Covered soil	CK	6254.2 ± 150.0e	457.3 ± 9.3e	39.4 ± 0.9de	47.2 ± 1.1bc
	T_C_	7494.0 ± 235.6a	595.3 ± 7.4a	41.5 ± 1bc	49.4 ± 0.1a
	T_B_	7257.3 ± 158.2ab	502.3 ± 11.8c	43.3 ± 1.2a	48.3 ± 0.7ab
	T_J_	7102.6 ± 197.5bc	513.3 ± 8.5c	40.3 ± 0.5cd	48.4 ± 1ab
	T_J+B_	7174.8 ± 182.2bc	543.0 ± 15.7b	40.6 ± 1.1cd	47.9 ± 0.5ab
	T_C+B_	6760.0 ± 146.8d	455.0 ± 4.4e	38.7 ± 0.8e	47.3 ± 1.1bc
	T_J+50%C_	6929.0 ± 194.0cd	475.5 ± 10.5d	41.6 ± 1.1bc	46.3 ± 0.9c
	T_J+B+50%C_	7357.7 ± 133.6ab	540.7 ± 13.3b	42.2 ± 0.7ab	47.6 ± 0.9bc
	Mean	7041.2 ± 174.8a	510.3 ± 10.1a	41.0 ± 0.4a	47.9 ± 0.4a
F-value	S	116.09**	42.51*	202.65**	0.01ns
	T	33.07**	59.20**	8.24**	4.64**
	S×T	2.53*	37.89**	13.29**	2.18ns

Different lowercase letters for the same indicator indicate significant differences among treatments (P < 0.05). Asterisks above the values indicate significant differences among treatments: *, P < 0.05; **, P < 0.01; ***, P < 0.001; ns, not significant.

In 2024–2025, soil-covered sowing increased mean yield by 4.3%, effective spike number by 1.9%, grains per spike by 12.5%, and thousand-grain weight by 4.0%. T_C_ produced the highest yield under both uncovered and soil-covered sowing, exceeding the respective CK values by 19.6% and 12.5%. Under soil-covered sowing, T_B_ and T_J+B_ also produced significantly higher yields than CK. For T_C_, the yield advantage coincided with 589.5 × 10^4^ effective spikes ha^–1^ and 43.7 grains per spike.

In 2025–2026, soil-covered sowing increased mean yield by 5.2%, effective spike number by 3.5%, and grains per spike by 8.2%, whereas thousand-grain weight changed only slightly. Under uncovered sowing, T_J+B+50%C_ and T_C_ increased yield by 21.8% and 21.3% relative to CK, respectively. T_J+B+50%C_ produced the highest grains per spike, T_C+B_ the highest thousand-grain weight, and T_B_ the highest effective spike number. Under soil-covered sowing, T_C_ produced the highest yield, 19.8% above CK, while T_J+B+50%C_ and T_B_ also significantly exceeded CK. T_C_ had the highest effective spike number and a thousand-grain weight of 49.4 g, whereas T_B_ had the highest grains per spike. Across both years, T_C_ was the highest-yielding treatment under soil-covered sowing. The contributions of individual yield components varied among treatments, sowing methods, and years.

### 3.6 Correlations among germination, emergence, seedling growth, and yield traits

Under uncovered sowing, germination and seedling vigor indices (GP, GR, GI, SVI, and VI) were positively correlated with one another (**Figure 7A**). Emergence timing indices (t25, t50, t75, and t99) were also positively correlated with one another. These indices were generally negatively correlated with the emergence-rate indices Vmax, V25–75, and MER. Effective spike number was positively correlated with Vmax, V25–75, MER, UI, and several seedling growth traits. Grain yield was positively correlated with CGR, G, RGR, LWR, and LAI. Under soil-covered sowing, the overall correlation pattern was similar (**Figure 7B**). Grain yield was positively correlated with GP, GI, SVI, VI, and several seedling growth traits. It was negatively correlated with the emergence timing indices t50, t75, and t99. Effective spike number was positively correlated with Vmax, V25–75, MER, UI, CGR, G, RGR, and LAI. Across both sowing methods, higher germination and seedling vigor, faster and more uniform emergence, and stronger canopy growth were associated with higher grain yield and effective spike number. Conversely, longer emergence times were associated with lower values of yield-related traits. These correlations describe coordinated variation among early establishment traits and yield components but do not establish causal relationships.

**Figure 7:**
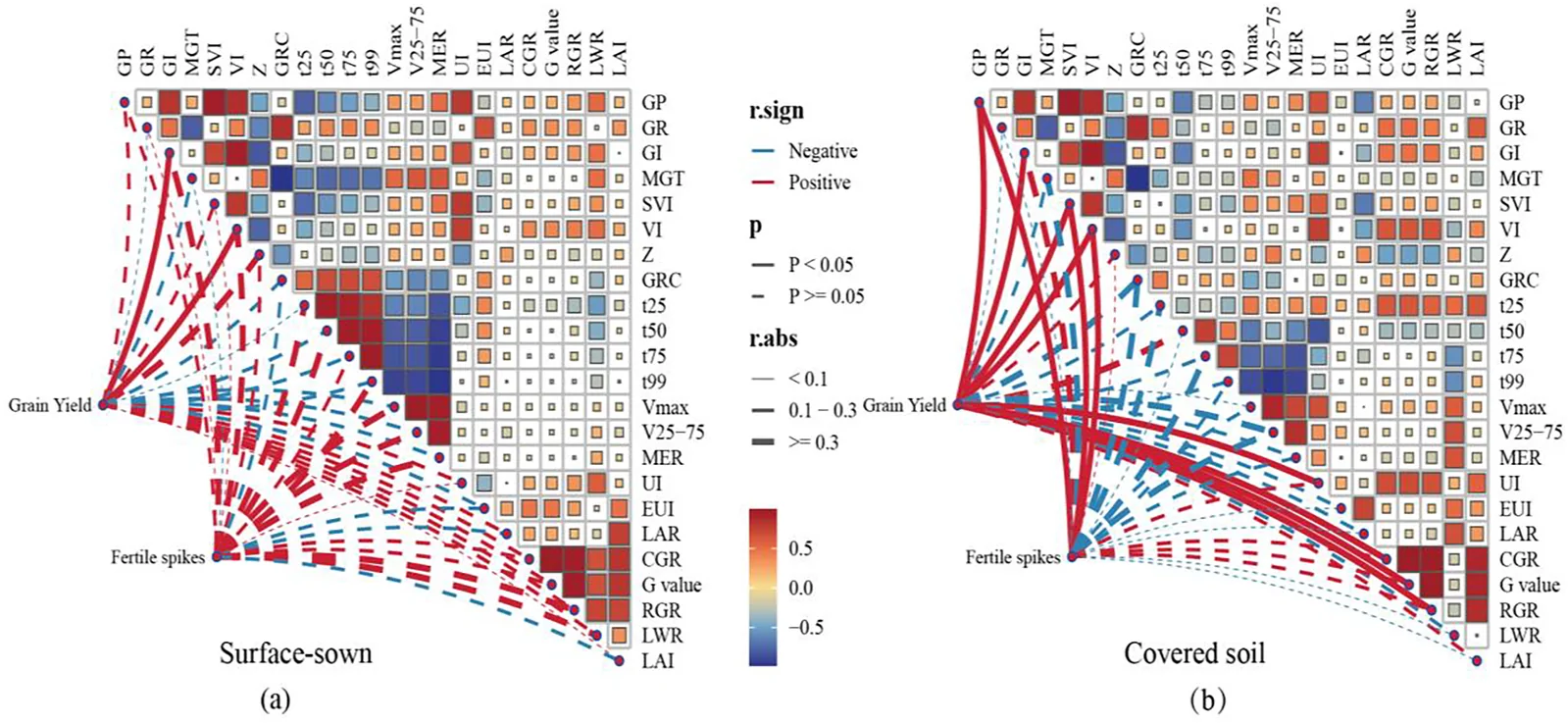
Correlation analysis of germination traits, emergence dynamics, seedling growth traits, grain yield, and effective spike number under **(A)** uncovered and **(B)** soil-covered sowing. Line color denotes the direction of the correlation (r.sign): red and blue lines represent positive and negative correlations, respectively. Line type (solid or dashed) denotes the statistical significance category based on the corresponding P value. The parameter r.abs denotes the absolute value of the correlation coefficient, |r|.

## 4 Discussion

### 4.1 Coordinated effects of seed-dressing treatments on germination and early stand establishment

In rice–wheat rotation systems, successful wheat establishment involves germination, field emergence, root development, and early stand formation. These processes represent distinct but interrelated stages. Germination indices reflect seed performance, emergence traits describe field establishment, and shoot and root traits characterize post-emergence growth (Cañizares et al., 2025; Hoseini et al., 2022; Manschadi et al., 2006; Slafer et al., 2014). In practice, seeding rates should account for laboratory germination percentage, thousand-kernel weight, and a field emergence correction factor to achieve the target plant density. This correction factor depends on soil moisture, seedbed quality, and sowing depth, linking laboratory seed performance to expected field establishment. Their combined evaluation is particularly important in rice-stubble fields, where delayed sowing and variable soil moisture may restrict uniform stand establishment. Seed priming and seed dressing can improve germination and seedling establishment under unfavorable conditions (Afzal et al., 2020; Finch-Savage and Bassel, 2016; Tursynova et al., 2025; Javed et al., 2021; Wang and Shi, 2024). However, treatment efficacy is often evaluated using individual traits or measurements from a single developmental stage. In this study, T_C_ showed favorable responses in several establishment and growth traits, although these responses were not uniform across all indices and growing seasons. T_B_ was associated with faster field emergence. The effects of T_J+B_ based treatments were more dependent on sowing conditions. These results support evaluating seed-dressing treatments across successive establishment stages rather than relying on germination percentage or a single-season yield response (Javed et al., 2021; Elhaj Baddar and Unrine, 2018; Wasson et al., 2012). The coordinated responses observed under T_C_ suggest that its agronomic performance may be associated with several components of early establishment. Greater root development and activity could be associated with improved access to soil resources and shoot growth. However, nutrient uptake, reserve mobilization, hormonal regulation, and stress tolerance were not measured directly. These mechanisms should therefore be regarded as hypotheses. Likewise, correlations between early traits and grain yield indicate coordinated responses but do not establish causality.

### 4.2 Sowing method modulates the effects of seed-dressing treatments

Sowing method substantially influenced emergence dynamics and treatment performance. Soil covering shortened emergence time and improved emergence rate and uniformity, indicating that the physical sowing environment strongly affected stand establishment. Improved seed–soil contact and moisture availability may have contributed to these responses, but neither factor was measured directly. The significant interaction between sowing method and seed-dressing treatment shows that formulation performance cannot be generalized across sowing conditions. T_B_ was associated with faster emergence, particularly under uncovered sowing. In contrast, T_J_ and T_J+50%C_ showed no consistent emergence advantage under soil-covered sowing. This context dependence agrees with previous reports that seed-treatment responses vary with active ingredient, application rate, crop genotype, and soil environment (Paparella et al., 2015; Farooq et al., 2019). Although reserve mobilization and pre-germination metabolic activation may contribute to priming responses (Javed et al., 2022; Elhaj Baddar and Unrine, 2018), these processes were not quantified in the present study. T_C_ showed favorable responses across seedling biomass, growth indices, chlorophyll content, root activity, and root architecture under several of the tested conditions. Coordinated shoot and root development may benefit wheat establishment under challenging field conditions (Wasson et al., 2012; Manschadi et al., 2006). Nevertheless, the significant treatment × sowing method interaction indicates that these benefits depend on the establishment environment. Soil covering improved baseline emergence conditions, whereas seed-dressing treatments provided formulation-specific responses. Treatment selection should therefore consider both the principal establishment constraint and the intended sowing method.

### 4.3 Relationships between early establishment and yield formation

Early establishment advantages were not consistently translated into grain yield across treatments and growing seasons. T_C_ produced the highest grain yield under soil-covered sowing in both seasons and maintained relatively high performance under uncovered sowing. Its yield advantage was mainly associated with effective spike number, although grains per spike and thousand-grain weight also contributed under some conditions. These responses are consistent with the recognized roles of these components in wheat yield formation (Hoseini et al., 2022; Ben-Jabeur et al., 2019; Finch-Savage and Bassel, 2016) and with evidence linking pre-anthesis growth to grain number (Slafer et al., 2014). The association between effective spike number and grain yield provides a possible link between stand establishment and final productivity. Improved emergence and early growth may enhance seedling survival and productive tiller formation. However, this pathway was not tested directly, and treatment effects during later developmental stages may also have contributed. The relationship should therefore be interpreted as an association rather than a demonstrated causal mechanism. The other treatments showed narrower or less stable yield responses. T_J+B+50%C_ achieved a high yield under uncovered sowing in one season, whereas T_B_ combined rapid emergence with favorable yield performance under some soil-covered conditions. Similar improvements in emergence, chlorophyll content, yield components, and grain yield have been reported for zinc, biostimulant, and microbial seed treatments (Zhang et al., 2007; Reed et al., 2022). The present results further indicate that such responses depend on the sowing environment and may involve different yield components. Within this single-site study involving one cultivar over two growing seasons, T_C_ showed promising performance across establishment and yield traits. T_B_ improved emergence, suggesting potential value where delayed or uneven emergence is a concern. The yield response to T_J+B+50%C_ under uncovered sowing was observed in one season and does not establish a consistent benefit under this sowing method. Before general treatment recommendations can be made, these findings require validation across cultivars, locations, and additional growing seasons, with attention to performance across developmental stages and economic feasibility.

### 4.4 Limitations and future perspectives

This study evaluated one wheat cultivar at a single loam-soil site in Sichuan Province over two growing seasons, limiting its generalizability. Data were analyzed separately by season because seeding rates differed, confounding seasonal and seeding-rate effects; season × treatment interactions were therefore not assessed. Commercial formulations were tested as complete products, preventing attribution of their effects to individual components, rates, or interactions. Coolas contains thiamethoxam, fludioxonil, and difenoconazole, but disease and pest pressure were not monitored. Protective effects therefore could not be distinguished from direct effects on growth or waterlogging tolerance. The proposed physiological mechanisms remain unverified, and the benefits of T_C_, T_B_, and T_J+B+50%C_ depended on sowing method. Economic outcomes were not assessed.

Future studies should include multiple sites, seasons, cultivars, and establishment conditions, with seeding rates standardized or included as an experimental factor. Factorial experiments combining disease and pest monitoring with physiological measurements are needed to separate formulation effects and test the proposed mechanisms. Assessments of agronomic stability, environmental safety, and economic returns would further support region-specific seed-dressing recommendations.

## 5 Conclusions

This study evaluated whether seed-dressing treatments could improve wheat establishment under contrasting sowing conditions in a rice–wheat rotation system. Across two growing seasons, selected treatments improved establishment and increased grain yield, although responses varied with treatment composition and sowing method. Soil-covered sowing generally advanced emergence and altered treatment performance. T_C_ and T_J+B+50%C_ showed favorable responses across several measured traits. T_C_ increased grain yield under both sowing methods in both seasons, while TJ+B+50%C showed improvements in germination, root development, and grain yield in 2025–2026. Soil covering further promoted rapid and uniform establishment and modified treatment performance, highlighting the importance of matching seed-dressing strategies to specific sowing environments. Collectively, these findings demonstrate the potential of seed dressing to improve wheat performance under the tested conditions and highlight the importance of evaluating responses from germination through yield formation. However, because this study evaluated a single wheat cultivar at one location over two growing seasons, the agronomic stability and broader applicability of these treatments require further validation across cultivars, environments, and production years, together with assessments of their economic feasibility.

## Data Availability

The original contributions presented in the study are included in the article/supplementary material. Further inquiries can be directed to the corresponding author.
